# Childhood pemphigus vulgaris is a challenging diagnosis

**DOI:** 10.4322/acr.2021.267

**Published:** 2021-04-30

**Authors:** Gastão Tenório Lins, Nathalia Lages Sarmento Barbosa, Eulina Maria Vieira de Abreu, Klinger Vagner Teixeira da Costa, Kelly Chrystine Barbosa Meneses, Rodrigo Neves Silva, Sonia Maria Soares Ferreira

**Affiliations:** 1 Centro Universitário CESMAC, Curso de Odontologia, Maceió, AL, Brasil; 2 Posto de Atendimento Médico Salgadinho-Serviço de Dermatologia, Maceió, AL, Brasil; 3 Centro Universitário CESMAC, Curso de Medicina, Maceió, AL, Brasil; 4 Centro Universitário CESMAC, Mestrado Profissional Pesquisa em Saúde, Maceió, AL, Brasil; 5 Centro Universitário CESMAC, Laboratório de Patologia oral, Maceió, AL, Brasil; 6 Posto de Atendimento Médico Salgadinho-Serviço de Estomatologia, Maceió, AL, Brasil

**Keywords:** Pemphigus, Child, Fluorescent Antibody Technique, Direct

## Abstract

Pemphigus Vulgaris (PV) is an uncommon autoimmune and blistering mucocutaneous disease. Childhood Pemphigus Vulgaris (CPV) is a pediatric variant of PV, which affects children below 12 years, being very rare among children under 10 years of age. CPV has similar clinical, histological, and immunological features as seen in PV in adults. The mucocutaneous clinical presentation is the most common in both age groups. Vesicles and erosions arising from the disease usually cause pain. A few CPV cases have been reported in the literature. This study reports a case of an 8-year-old male patient with oral lesions since the age of 3 years, and the diagnosis of pemphigus was achieved only 2 years after the appearance of the initial lesions. CPV remains a rare disease, making the diagnosis of this clinical case a challenge due to its age of onset and clinical features presented by the patient. Therefore, dentists and physicians should know how to differentiate CPV from other bullous autoimmune diseases more common in childhood.

## INTRODUCTION

Pemphigus Vulgaris (PV) is an uncommon autoimmune blistering disease involving the skin and mucous membranes.^1^^,^^2^ Most patients with PV have circulating autoantibodies against desmogleins 1 and 3, which are transmembrane proteins of the desmosomes. The clinical disorder is a consequence of the loss of cell adhesion, which occurs due to the attack on transmembrane proteins.^3^

Histopathological features are represented by intraepidermal acantholysis and intact basal layer.^1^ It is important to identify the level of bubble cleavage through histopathological examination to diagnose pemphigus and differentiate it from other subepidermal bullous lesions, as acantholytic keratinocytes can be observed in several vesiculobullous diseases (Hailey-Hailey-like Grover’s disease and Hailey-Hailey disease, among others).^4^

PV has a pediatric variant divided into juvenile PV (JPV) and childhood PV (CPV). There is no difference between PV and CPV concerning the diagnostic tests because CPV has similar clinical, histological, and immunological features seen in PV in adults.^2^^,^^3^

Among bullous diseases in childhood, CPV is the least frequent entity and thus can be misdiagnosed.^2^ The differential diagnosis of CPV includes (i) hand-foot-mouth disease, (ii) oral candidiasis, (iii) acute herpetic gingivostomatitis, (iv) erythema multiforme, (v) Behcet’s disease, (vi) lichen planus, and (vii) recurrent aphthous stomatitis.^5^

Clinical presentation includes flaccid blisters that produce erosions after rupture. Anamnesis should focus on duration, number of lesions, and recurrence of symptoms.^2^

Ancillary diagnostic methods include biopsy with immunofluorescence (IF) investigation.^2^ Direct immunofluorescence (DIF) identifies the presence of autoantibodies and complement fractions.^3^^,^^6^ CPV therapy is performed with topical or systemic steroids combined with adjuvant immunosuppressive drugs due to steroid-sparing effects.^3^^,^^6^

This study has the academic importance of reporting a case of CPV with aggressive clinical presentation in a male child.

## CASE REPORT

**Figure 1:**
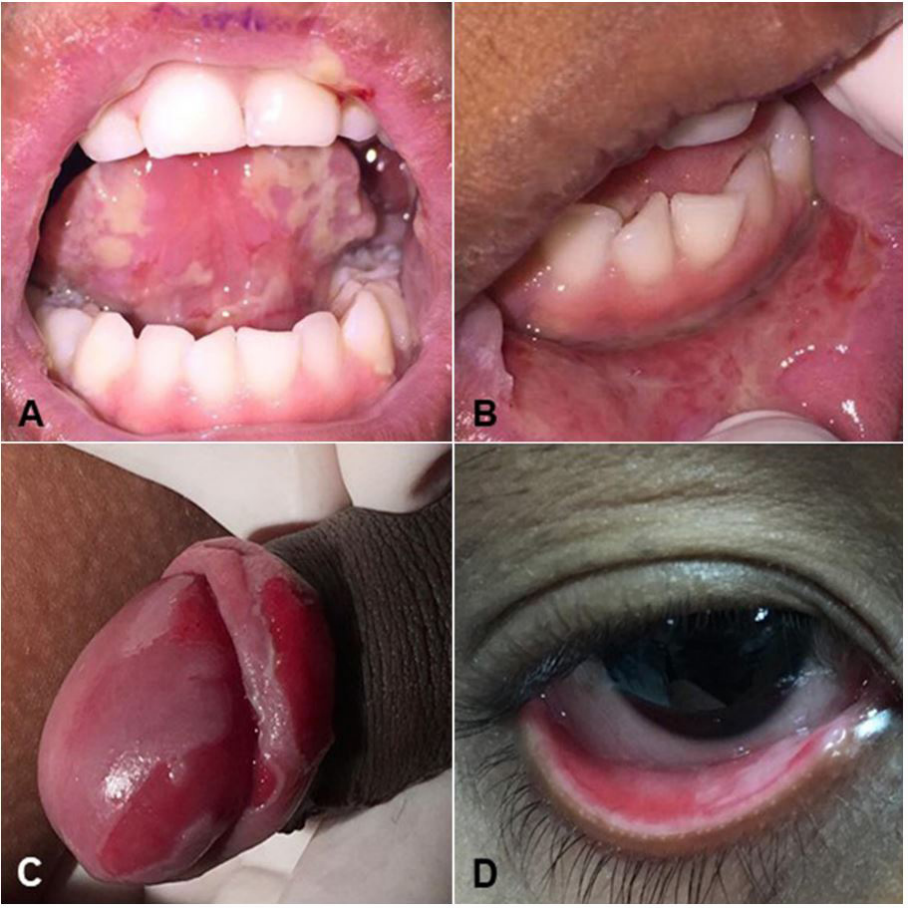
Lesions at the first appointment. **A** – Ulcerative lesions in the ventral surface of the tongue; **B** – Lesions with an ulcerative surface in lower labial mucosa (biopsy site); **C** – Ulcers in glans and foreskin; **D** – Ulcer in the mucosa of the lower eyelid.

An 8-years-old black male patient sought care in the stomatology clinic after looking for medical care without a definitive diagnosis and therapeutic success. His mother reported that the initial lesions appeared at the age of 3 years, initially in the tongue, then in other oral cavity areas. However, upon current examination, the patient presented mouth opening limitation, sialorrhea, and ulcers in the oral mucosa, glans, and lower eyelids, as well as urethral secretion. Oral lesions involved the upper and lower lip mucosa, the tongue’s ventral surface, hard palate, soft palate, oral commissures, lower alveolar mucosa, oral mucosa, and upper and lower gums (Figure 1).

**Figure 2:**
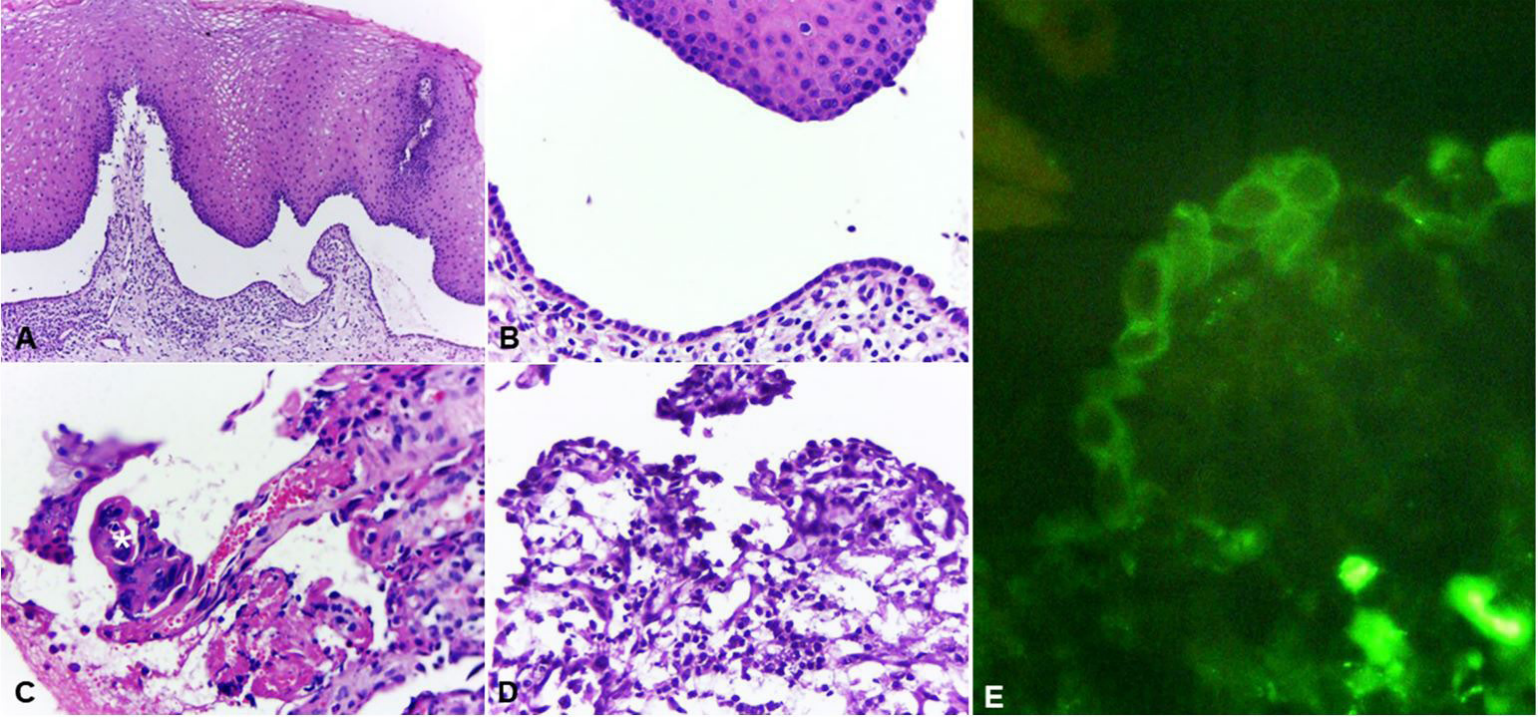
Photomicrographs of the biopsy specimen. **A** – Intraepithelial blister with a single layer of basal cells recovering the conjunctive tissue and lymphoplasmacytic inflammatory infiltrate with eosinophils (H&E, 5X); **B** – High magnification of image A (H&E, 20X); **C** – Ulcered area with grouped acantholytic cells (*) with degenerative alterations (H&E, 40X); **D** – View of the inferior area of epithelial blister and suprabasal slit showing tiny area with rare basal cells still attached to the conjunctive tissue (H&E, 40X); **E** – Positive direct immunofluorescence showing IgG3 in the intercellular space of cells of the remaining epithelium basal layer on the conjunctiva.

The patient also presented speech difficulties accompanied by hoarseness. Laryngoscopy showed ulcerations in the larynx and pharynx. Incisional biopsy in the lower labial mucosa was performed. The histopathological report showed PV and DIF confirmed the diagnosis with positive detection of intercellular IgG3 (Figure 2).

Initial treatment comprised prednisone (1mg/kg/day) and Dapsone (1 mg/kg/day). Six months after treatment, only oral lesions remained, but the clinical condition worsened the next month, and lesions in the genital mucosa relapsed. At this moment, prednisone (1mg/kg/day) and dapsone (1 mg/kg/day) were again prescribed until achieving clinical regression of genital lesions. With the eighth month of follow-up, ulcers were present only in the mouth and, in the ninth month of follow-up, the clinical mouth condition had improved, but lesions in the palate persisted. After a short time, ulcers in other sites reappeared.

When the patient completed one year and three months of treatment, the initial treatment, prednisone was gradually tapered to a maintenance dose of 12.5 mg daily, and dapsone was withdrawn due to side effects. At this moment, infection was diagnosed in the oral cavity and other sites of the patient's body. Thus sulfamethoxazole (200 mg) and trimethoprim (40 mg) BID were prescribed. This new therapeutical approach was applied to achieve clinical regression of the lesions.

To date, the patient of this clinical case presented marked weight gain and growth retardation, even with the use of a steroid-sparing drug and a decrease in steroid dosage.

**Figure 3:**
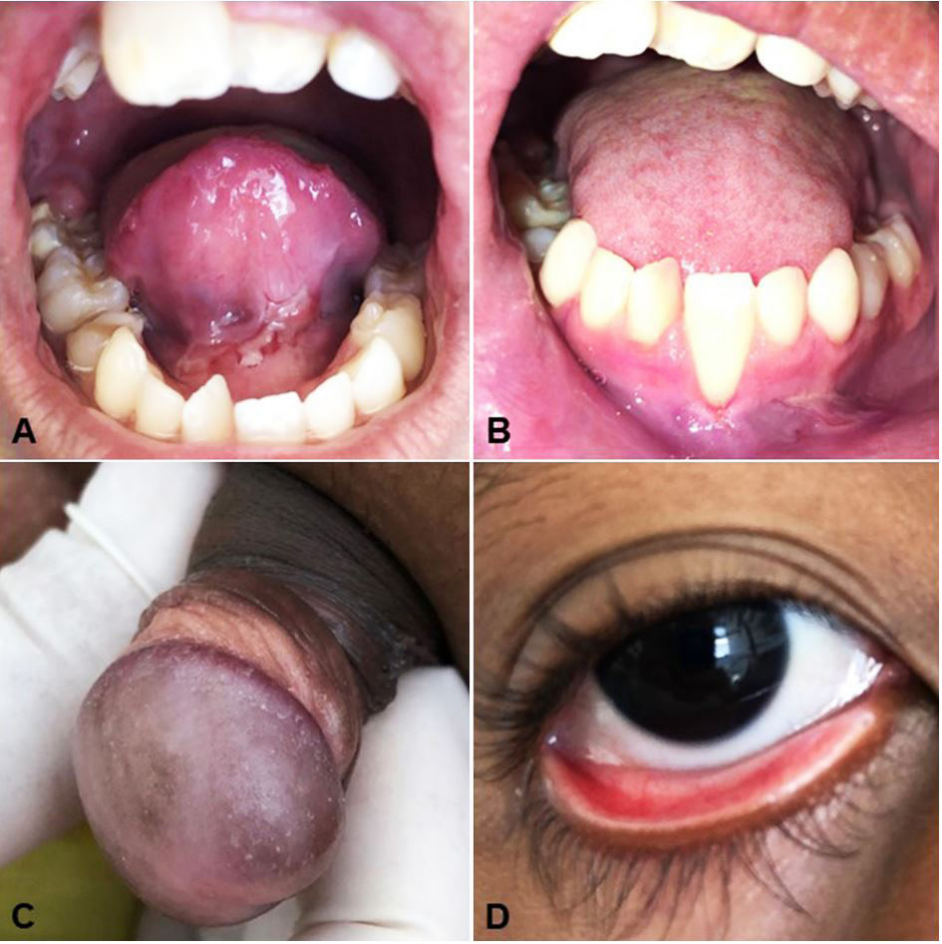
Lesions on the last follow-up. **A** – Ulcerative lesions in the ventral surface of the tongue; **B –** Ulcers in the transition between alveolar mucosa and lower labial mucosa; **C –** Remission of lesions in glans and foreskin; **D** – Absence of lesion in the lower eyelid.

In the last follow-up, lesions in the eyelids, larynx, pharynx, and glans had disappeared without relapse. However, ulcers of the oral cavity remain in the lower labial mucosa and ventral surface of the tongue (Figure 3). In addition to systemic medications, topical corticosteroid on the remaining oral lesions was added to the therapeutic regimen. The patient currently completed a clinical follow-up of 2 years and 10 months, and his clinical status substantially improved.

## DISCUSSION

This article describes a case of CPV with aggressive behavior. CPV accounts for approximately 1.4-3.7% of all PV cases. It is rare in children under 10 years of age. The age of onset comprises children under the age of 12 years, being lower in boys than in girls.^1^^-^^3^^,^^5^^,^^7^^,^^8^ Pemphigus in children younger than 12 years is known as childhood pemphigus, and in those aged between 12-18 years as juvenile pemphigus.^7^ PV in children remained a rare disease since 1955 when the first case of PV in the pediatric age was reported. Since then, approximately 50 new cases have been reported to date.^9^ Interestingly, our patient’s initial lesions appeared when he was 3 years old, and the definite diagnosis was made 2 years after the first clinical signs.

Numerous vesicles and painful erosions in the oral cavity clinically characterize PV. One of the first symptoms is intense pain associated with difficulty in defecating, urinating, and ingesting foods and liquids.^3^^,^^6^ The painful symptomatology in this patient remained absent throughout the clinical course of the disease, allowing the child to feed and urinate normally, unlike most clinical cases reported in the literature.

The etiology of pemphigus vulgaris is uncertain. In some cases, it may have a strong genetic basis as it has been more frequently reported in certain racial groups. Although the exact etiology remains obscure, a wide range of antigenic factors, including drugs, herpes virus and bacterial infections, and malignancy, have been suggested as triggering factors.^10^^,^^11^^,^^13^

The most frequently affected areas include the skin or mucous membranes of the oral cavity, anus, conjunctiva, and genital areas. An important difference between adult and pediatric PV is the high incidence of genital and ocular involvement in the pediatric variant.^3^ In addition, CPV has a more variable clinical course when compared to PV in adults.^3^^,^^4^ When the pharynx and larynx are involved, hoarseness may be present.^1^ In this case, lesions affected the same mucosa areas described in the literature. Due to the presence of hoarseness among our patient’s clinical features, a laryngoscopy was performed and showed the presence of ulcers in the larynx and pharynx.

The majority of patients with CPV have lesions in both mucosa and skin, with the appearance of oral lesions being the first manifestation of the disorder.^3^^,^^4^ Ulcers in the oral mucosa were the first sign of the disease of this case report, followed by the mucous membranes of the conjunctiva and glans. However, skin lesions did not appear at any stage of the disease.

According to Surya et al.,^2^ oral lesions usually affect gingiva, oral mucosa, lips, hard and soft palate.^2^ In addition to these areas, oral lesions also affected the ventral surface of the tongue and lower alveolar mucosa. These authors also reported that the diagnosis could be performed either by routine histopathology or DIF, but the recommendation is to perform both.

The differential diagnosis of PV in children includes entities affecting the mucocutaneous tissues or only the mucous membranes, some affecting only the oral cavity. The mucocutaneous dermatological diseases include (i) bullous epidermolysis, (ii) linear IgA disease, (iii) paraneoplastic pemphigus, (iv) erythema multiforme, (v) cicatricial pemphigoid, (vi) erosive lichen planus.^14^

Recurrent aphthous stomatitis and acute herpetic gingivostomatitis present signs only in the oral mucosa. There is the appearance of ulcers with a yellowish base in the former, surrounded by an erythematous halo, with regular margins and disappearance without treatment, which characterizes an acute course. The second one is characterized by small yellowish vesicles that break quickly, giving rise to ulcers with an erythematous halo affecting the free and adherent gums.^14^ Knowing that the appearance of oral lesions is the first sign of PV in most patients, the physician needs to be acquainted with these diseases’ characteristics to differentiate them from PV, avoiding a delay in diagnosis.

Behçet's disease compromises the oral, genital and ocular mucous membranes.^14^ This disease was the main differential diagnosis of this clinical case because the patient attended the first consultation with lesions on these areas.

Biopsies performed on intact blisters provide better results, but these are rarely found since they rupture easily. When the specimen is taken from the center of the ulcerative lesion, it is histopathologically nonspecific. For precise DIF diagnosis and histopathological exam, biopsy has to be performed in the perilesional area.^2^^,^^10^ A single biopsy was performed in the perilesional area of the labial lesion in the patient of this case report. The biopsy specimen was split into two parts that were sent for histopathological and DIF evaluation.

When lesions are exclusively presented in the mucosa, IgG autoantibodies are directed against Dsg 3, an autoantigen of greater expression in the lower portions of mucosal squamous epithelium. DIF analysis makes it possible to observe an intercellular fluorescent IgG distribution, usually found in the lower layers of the epithelium. The method works due to an antigen-antibody reaction *in vitro*. Radiation-absorbing dyes (fluorochromes excited by ultraviolet) shine when tissue deposition of IgG occurs.^4^^,^^10^^,^^12^

There are three laboratory diagnostic tests related to antibodies. Direct Immunofluorescence is an exam considered as the “gold standard” for pemphigus diagnosis. The Indirect Immunofluorescence technique determines the presence of circulating autoantibodies in the serum and assists in diagnosis. Finally, the Immunohistochemical examination consists of a combination of immunological and histological methods for detecting specific antigens in tissues or cells (immunocytochemistry), based on the identification of the antigen-antibody complex. Markers for the detection of intercellular IgG and C3 can be used in PV. These last two techniques were not used because the main diagnostic hypothesis was PV since the first consultation of the patient with the specialist in Oral Medicine. Therefore, the biopsy was performed to the conventional histological analysis and DIF. These techniques were chosen to take into account recommendations by most authors.^4^^,^^9^

Genital lesions have as differential diagnosis (i) sexual abuse, (ii) bullous fixed drug eruption, and (iii) bullous lichen sclerosis.^1^ At the patient’s first appointment, rapid tests were performed for Hepatitis B, Hepatitis C, HIV, and Syphilis, which showed negative results.

A delay in diagnosing CPV often occurs due to the rarity of PV in this age group and similar clinical appearance with other bullous and ulcerative diseases that affect the oral cavity. In this context, a brief history and clinical examination can lead to incorrect diagnosis and, consequently, to inadequate treatment.^2^^,^^5^ A recent case report also reported a delay in diagnosing PV in a child. The authors reported that it is possible due to the wide variety of blistering diseases most prevalent and the lack of familiarity with this entity.^9^ The patient in this case report had only oral lesions when he was initially evaluated by a physician, a fact that may have hindered the diagnosis. It also emphasizes the importance of the differential diagnosis of CPV.

The basis of PV treatment is systemic corticosteroids, as they present potent anti-inflammatory and immunosuppressive action. The most frequently used oral steroids for PV treatment is prednisone, followed by prednisolone. Most authors prefer the administration of full doses (1 to 2 mg/ kg/ day) since the beginning of the therapy, thus avoiding progressive dose increase.^4^ In children, the dose should be chosen according to age, weight, disease severity, and drug side-effects. Prednisolone dosage is adjusted according to the clinical response and slowly reduced in cases showing improvement.^5^^,^^13^

Immunomodulating drugs can also be used as steroid-sparing agents due to common side effects after prolonged use of corticosteroids and enhance therapeutic response. The search for adjunctive treatments with immunomodulators was encouraged to reduce the dose and duration of treatment with corticosteroids. A common steroid-sparing drug used in pemphigus therapy is dapsone. Drugs can be stopped when lesions disappear, but, usually, in pediatric patients, pemphigus shows a relapsing course as observed in adults, and total remission is rare. Therefore, maintenance dose (5–20 mg/dl) may be required in some patients.^2^^,^^5^^,^^13^ In the present case report, dapsone was interrupted due to side effects. Dapsone is withdrawn due to its potential hematological adverse effects, such as methemoglobinemia, hemolysis, and agranulocytosis. These effects are mandatory and vary only in intensity.^15^ They are classified as dose-dependent effects. A study revealed that this drug was the most common cause of acquired methemoglobinemia, which is caused by hydroxylamine, a dapsone metabolite.^16^

Although sulfamethoxazole-trimethoprim is not a drug of treatment for pemphigus vulgaris, this drug was prescribed to our patient because he presented infection in different body sites. Sulfamethoxazole-trimethoprim was prescribed due to its ability to control superinfections, mainly by *Staphylococcus aureus*, and also due to the indirect immunomodulatory effect, regulating the secretion of IL-1 and TNF-α.^17^

Although there is scarce scientific evidence on the efficacy of different immunomodulatory drugs,^5^ satisfactory result was achieved in this case, even in the absence of the lesions’ complete remission. To support this statement, some facts must be considered: the complete CPV remission is rare, recurrences are common,^5^ and the patient's disease was misdiagnosed for 2 years.

Some patients are refractory to conventional therapy or become chronically steroid-dependent, so there is a need for new therapeutic options to treat pediatric PV.^1^ Since July 2018, rituximab was labeled by the US Food and Drug Administration (FDA) to refractive severe-to-medium pemphigus patients and adapted a year later by the European Committee health authorities.^8^^,^^18^ Thereby, a previous case report of a 14-year-old girl was treated with a modified two-dose regimen, performed 30 days apart, exceptionally due to financial reasons. This therapeutical approach proved highly efficient and long-standing, which encourages its further implementation.^8^ In addition, a case series concluded regarding the terms of efficacy and safety of rituximab. Increasing evidence supports that rituximab is a good treatment choice, not only in adults but also in pediatric patients with pemphigus.^18^ The treatment with rituximab was not applied in the index case due to financial restraints.

Due to the patients’ growth and development in childhood, the frequency of side effects of the high doses and prolonged use of steroids is higher in children than in adults. The most common are growth retardation, infection, obesity, social and psychological distress.^1^^,^^3^^,^^6^

Despite the use of numerous immunosuppressive therapies and a better prognosis of CPV concerning PV, some patients are refractory to conventional treatment, as occurred in this case report, or may become steroid-dependent. Therefore, new therapeutic options for pediatric PV are expected.^1^^,^^3^ In the last years, some case reports described that the rituximab can be considered an effective adjuvant therapy when treating resistant vesiculobullous disorders in pediatric patients. However, a greater number of patients and long-term follow-up is required to propose a definite conclusion.^1^^,^^6^^-^^8^^,^^18^^,^^19^

## CONCLUSION

In conclusion, CPV diagnosis is still challenging due to the varied clinical features and the clinical similarity to other pediatric autoimmune bullous diseases. These characteristics were observed in this case report, which presented extensive painless lesions at different sites of mucous membranes and late diagnosis. It demonstrates the importance of reporting new CPV cases. A better understanding of CPV characteristics, such as the fact that oral involvement can be the first signal of the disease and how to diagnose it, enables the correct diagnosis, leading to earlier detection of the disease and indication of appropriate therapy.
